# Therapeutic Targeting of Proteostasis in Amyotrophic Lateral Sclerosis—a Systematic Review and Meta-Analysis of Preclinical Research

**DOI:** 10.3389/fnins.2020.00511

**Published:** 2020-05-25

**Authors:** Elizabeth Elliott, Olivia Bailey, Fergal M. Waldron, Giles E. Hardingham, Siddharthan Chandran, Jenna M. Gregory

**Affiliations:** ^1^UK Dementia Research Institute, The University of Edinburgh, Edinburgh, United Kingdom; ^2^Centre for Clinical Brain Sciences, The University of Edinburgh, Edinburgh, United Kingdom; ^3^The Anne Rowling Regenerative Neurology Clinic, The University of Edinburgh, Edinburgh, United Kingdom; ^4^The Euan MacDonald Centre, University of Edinburgh, Edinburgh, United Kingdom; ^5^Edinburgh Neuroscience, The University of Edinburgh, Edinburgh, United Kingdom; ^6^MRC Edinburgh Brain Bank, Academic Department of Neuropathology, The University of Edinburgh, Edinburgh, United Kingdom; ^7^Ashworth Laboratories, Institute of Evolutionary Biology and Centre for Immunity Infection and Evolution, University of Edinburgh, Edinburgh, United Kingdom; ^8^Centre for Discovery Brain Sciences, The University of Edinburgh, Edinburgh, United Kingdom; ^9^Centre for Brain Development and Repair, inStem, Bangalore, India; ^10^MRC Centre for Regenerative Medicine, The University of Edinburgh, Edinburgh, United Kingdom; ^11^Edinburgh Pathology, The University of Edinburgh, Edinburgh, United Kingdom

**Keywords:** systematic review, meta-analysis, amyotrophic lateral sclerosis, motor neurone disease, proteostasis, preclinical, therapeutic, survival

## Abstract

**Background:** Amyotrophic lateral sclerosis (ALS) is a rapidly progressive fatal neurodegenerative condition. There are no effective treatments. The only globally licensed medication, that prolongs life by 2–3 months, was approved by the FDA in 1995. One reason for the absence of effective treatments is disease heterogeneity noting that ALS is clinically heterogeneous and can be considered to exist on a neuropathological spectrum with frontotemporal dementia. Despite this significant clinical heterogeneity, protein misfolding has been identified as a unifying pathological feature in these cases. Based on this shared pathophysiology, we carried out a systematic review and meta-analysis to assess the therapeutic efficacy of compounds that specifically target protein misfolding in preclinical studies of both ALS and FTD.

**Methods:** Three databases: (i) PubMed, (ii) MEDLINE, and (iii) EMBASE were searched. All studies comparing the effect of treatments targeting protein misfolding in pre-clinical ALS or FTD models to a control group were retrieved.

**Results:** Systematic review identified 70 pre-clinical studies investigating the effects of therapies targeting protein misfolding on survival. Meta-analysis revealed that targeting protein misfolding did significantly improve survival compared to untreated controls (*p* < 0.001, df = 68, α = 0.05, CI 1.05–1.16), with no evidence of heterogeneity between studies (*I*^2^ = 0%). Further subgroup analyses, evaluating the effect of timing of these interventions, showed that, only treating prior to symptom onset (*n* = 33), significantly improved survival (*p* < 0.001, df = 31, α = 0.05, CI 1.08–1.29), although this likely reflects the inadequate sample size of later time points. Furthermore, arimoclomol was found to significantly reduce secondary outcome measures including: (i) histological outcomes, (ii) behavioral outcomes, and (iii) biochemical outcomes (*p* < 0.005).

**Conclusions:** This analysis supports the hypothesis that protein misfolding plays an important role in the pathogenesis of ALS and FTD and that targeting protein misfolding, at least in pre-clinical models, can significantly improve survival, especially if such an intervention is administered prior to symptom onset.

## Introduction

Amyotrophic lateral sclerosis (ALS) is a progressive multi-system neurodegenerative disease caused by the selective degeneration of brain and spinal cord motor neurons. The condition is markedly heterogeneous with respect to both the genetic and pathologic basis and the phenotypic manifestations. Survival ranges from only a few months to decades. The motor manifestations of the condition also vary widely according to both the site of onset and the rate of disease progression (Pupillo et al., 2014; Martin et al., 2017). It is now known that 50% of ALS patients are also affected by cognitive impairment and that the criteria for co-morbid dementia in ALS-Frontotemporal dementia (ALS-FTD) is met in 10–15% of cases (Goldstein and Abrahams, 2013; Crockford et al., 2018). The identification of a misfolded protein (TDP−43), common to both ALS and FTD has redefined these diseases as existing on a clinico-pathological spectrum (Arai et al., 2006; Neumann et al., 2006). TDP-43 proteinopathy is identified at post mortem in almost all cases of ALS and up to 50% of FTD cases (Neumann et al., 2006; Cairns et al., 2018). While ALS disease causing mutations have now been identified in over 25 genes involved in biological processes ranging from transcriptional regulation, energy metabolism to proteostasis (Nguyen et al., 2018), mutations in only four genes account for over 50% of all familial ALS cases (Ling et al., 2013). The corresponding protein products or associated proteins for these four genes; *TDP-43, SOD1, FUS* and *C9orf72* have been identified in pathological ubiquitinated aggregates across mutant and sporadic cases alike (Ramesh and Pandey, 2017).

Transactivation response DNA binding protein 43 kDa, TAR DNA-binding protein 43 (TDP-43), the neuropathological hallmark of ALS (Arai et al., 2006; Neumann et al., 2006) is a ubiquitously expressed DNA and RNA binding protein from the heterogeneous nuclear ribonucleoprotein (HnRNP) family, which regulates transcription and splicing. The full length 414 amino acid protein contains an N-terminal domain, two RNA-recognition motifs (RRM) and a glycine rich C-terminal sequence (Ayala et al., 2005). In cases of ALS and FTD, TDP-43 is truncated in to c-terminal fragments (25 and 35 kDa in size), hyperphosphorylated, ubiquitinated and mislocalised from the nucleus forming cytoplasmic aggregates (Neumann et al., 2006; Van Deerlin et al., 2008). Fused in sarcoma (FUS), is another ubiquitously expressed HnRNP protein and consists of an N-terminal low complexity domain, RGG-rich domains, a RRM, a zinc finger domain and a nuclear localization signal [(Monahan et al., 2017); NLS]. FUS pathology was identified within a subgroup of FTD cases (Neumann et al., 2009) and subsequently FUS cytoplasmic inclusions have been identified in human neuronal and glial cells from spinal cord tissue of ALS cases with known *FUS* mutations, sporadic ALS, ALS/FTD and non-*SOD1* familial ALS cases (Deng et al., 2010). FUS mislocalisation affects approximately 5% of familial cases of ALS and fewer than 1% of sporadic cases. This is in stark contrast to TDP-43 inclusions, which are present in the majority of cases of ALS. Whilst FUS protein inclusions do not co-occur in the presence of TDP-43 inclusions, both TDP-43 and FUS proteins contain a highly aggregation prone domain with a high density of exposed hydrophobic residues. Mutations within this aggregation-prone region further increase the hydrophobicity and thus its tendency to aggregate (Patel et al., 2015).

Copper-zinc superoxide dismutase protein (SOD1) is a dimeric anti-oxidant enzyme, the pathological neuronal and glial aggregates of which have also been identified in post mortem sporadic and familial ALS cortical and spinal cord tissue (Shibata et al., 1996; Banci et al., 1998; Valentine et al., 2005; Forsberg et al., 2010, 2019). *SOD1* mutations have been found to destabilize the protein structure increasing hydrophobic surface exposure thereby promoting aggregation (Münch and Bertolotti, 2010; Tompa and Kadhirvel, 2019). Other factors influencing the propensity of wild type protein to aggregate have been studied including oxidative damage or metalation status which may account for sporadic cases (Watanabe et al., 2001; Rakhit et al., 2004; Tompa and Kadhirvel, 2019). The commonest cause of ALS/FTD, the *C9orf72* hexanucleotide repeat expansion has also been identified as a prominent proteinopathy (Al-Sarraj et al., 2011). This hexanucleotide repeat has been found to lead to non-ATG translation producing highly aggregation-prone dipeptide repeat (DPR) proteins (Ash et al., 2013). Concomitant TDP-43 aggregates are also a prominent part of *C9orf72* pathology found at post mortem. Additionally, TDP-43 negative inclusions comprised of DPRs as well as markers of the ubiquitin proteasome system (UPS) have been identified in FTD and ALS cases with a *C9orf72* mutation (Mackenzie et al., 2013). Accumulation of p62 otherwise known as Sequestosome 1 (SQSTM1), a ubiquitin binding protein and adapter molecule for autophagy is also a pathological feature of neuronal and glial inclusions across a broad range of neurodegenerative diseases (Kuusisto et al., 2001), however extra motor p62 neuronal and glial inclusions (in particular the granule cell layers of the cerebellum and hippocampus) have been found to be over-represented in *C9orf72* cases (Al-Sarraj et al., 2011; Cooper-Knock et al., 2012).

Proteostasis consists of parallel complementary mechanisms operating to continuously reduce the burden of intra- and extracellular protein misfolding. The main intracellular systems are the ubiquitin proteasome system (UPS) and autophagy where the UPS encompasses the initial tagging of proteins with ubiquitin molecules followed by degradation by the 26S proteasome complex (Glickman and Ciechanover, 2002). Autophagy is a lysosomal degradation system and includes the subtypes; macroautophagy to degrade large protein aggregates and damaged organelles, microautophagy as the direct uptake of cytosolic components in to the lysosome and chaperone-mediated autophagy (Guo et al., 2018). Chaperone-mediated autophagy, is the major pathway for maintaining both intra- and extracellular proteostasis and involves the formation of target protein-chaperone complexes which are then transported in to the lysosome (Glick et al., 2010). The heat shock proteins (HSPs) as chaperone proteins are also involved in protein maintenance more broadly; facilitating normal protein folding as well as monitoring protein quality (Kalmar and Greensmith, 2017). Over-arching regulatory signaling pathways are activated in response to an increased burden of misfolded proteins (ER stress) to increase the ER protein-folding capacity, termed the unfolded protein response (UPR). However, prolonged UPR activation has a paradoxical effect and correlates with cell death (Walter and Ron, 2011). Studies have demonstrated that the main ALS causative genes *TDP-43, SOD1, FUS* and *C9orf72* are each mechanistically linked to proteostatic systems (Kalmar and Greensmith, 2017). Increasingly other mutations associated with dysfunction of proteostatic mechanisms in ALS/FTD include either the proteasome and/or autophagy (*UBQLN2, TBK1, OPTN, VCP*) (Taylor et al., 2016).

Despite research efforts to trial ALS treatments for over two decades, an effective therapy for ALS is yet to be identified. Only one drug, the anti-glutamate treatment Riluzole, has been shown to confer a modest survival benefit and the search for novel therapies continues (Bensimon et al., 1994; Amyotrophic Lateral Sclerosis/Riluzole Study Group et al., 1996). One of the major barriers to the trialing of potential ALS therapies lies within the genetic and phenotypic heterogeneity of the disease, limiting the ability to conduct stratified and therefore statistically robust studies. The observation of protein misfolding as a unifying pathological feature across almost all cases of ALS and FTD despite the heterogeneity of these conditions presents a major therapeutic opportunity. A large body of research assessing the experimental effects of therapies which target and upregulate proteostatic mechanisms now exists. The primary aim of this systematic review and meta-analysis is to formally evaluate this literature to date and to assess the therapeutic potential of such interventions. A secondary aim of this work is to perform a structured quality assessment of the literature and to assess for publication bias to identify areas of improvement for future preclinical studies in this field.

## Materials and Methods

### Aims and Hypotheses

The aim of this piece of work was to assess the therapeutic potential of compounds tested in preclinical studies that target protein misfolding in ALS and FTD.

#### Primary Aim

1. Does targeting protein misfolding improve survival in ALS/FTD preclinical models?

#### Secondary Aims

1. Does the timing of intervention affect the efficacy of treatments targeting protein misfolding in ALS/FTD preclinical models?
2. Does targeting protein misfolding affect outcomes other than survival in ALS/FTD preclinical models?
3. Structured quality assessment of the included studies and assessment for publication bias

### PICOS Framework

**Population:** preclinical ALS/FTD animal models.

**Intervention:** therapeutics targeting protein misfolding.

**Comparison:** treatment vs. control group.

**Outcome measure:** primary outcome: mortality in animal models.

Secondary outcome measures: mean biochemical, histological and behavioral measurements.

**Study design:** all preclinical ALS or FTD animal or yeast model studies assessing the efficacy of therapies targeting proteostasis on survival compared to controls.

### Search Methods

Pre-clinical data were obtained, with no restrictions on publication date or language, from three databases; PubMed, MEDLINE and EMBASE using the following search terms (search date: 16/2/18). The references obtained from these searches were collated and imported onto Endnote, where duplicate studies were removed and full text articles retrieved. Language restrictions were applied and only articles written in plain English were included.

### Search Terms

#### PubMed

[(“motor neuron disease” OR “motor neuron” OR “MND” OR “ALS” OR “amyotrophic lateral sclerosis”) AND [(“protein misfolding”) OR (protein aggregation) OR (proteostasis)] AND (“mouse” OR “mice” OR “murine” OR “rat” OR “drosophila” OR “fruit fly” OR “c elegans” OR “zebra fish” OR “yeast”)]

#### MEDLINE

[(“motor neuron disease” OR “motor neuron” OR “MND” OR “ALS” OR “amyotrophic lateral sclerosis”) AND [(“protein misfolding”) OR (protein aggregation) OR (proteostasis)] AND (“mouse” OR “mice” OR “murine” OR “rat” OR “drosophila” OR “fruit fly” OR “c elegans” OR “zebra fish” OR “yeast”)]

#### EMBASE

[(“motor neuron disease” OR “motor neuron” OR “MND” OR “ALS” OR “amyotrophic lateral sclerosis”) AND [(“protein misfolding”) OR (protein aggregation) OR (proteostasis)] AND (“mouse” OR “mice” OR “murine” OR “rat” OR “drosophila” OR “fruit fly” OR “c elegans” OR “zebra fish” OR “yeast”)]

### Eligibility Criteria

Studies were included if they compared the effect of treatments targeting protein misfolding to a control group, in the specified animal or yeast model, in ALS or FTD. Studies were excluded if co-treatments were administered, if studies weren't investigating a therapeutic stated to target protein misfolding or didn't compare the treatment to a control group and didn't investigate therapeutics in ALS/FTD models or in the specified animal or yeast models. Human trials, reviews and case reports were also excluded.

### Screening

Studies were exported to SyRF, an online platform (http://syrf.org.uk) for performing systematic reviews where titles and abstracts were screened by two independent reviewers to determine their relevance. For studies that were disputed between the two reviewers a third reviewer assessed its relevance to reduce bias. The SyRF software is designed to limit learners bias by showing studies later in the screening process to one reviewer that had been screened early by the other reviewer.

### Data Extraction

The primary outcome measure was defined as mortality in animal models. The following data were extracted from included papers: paper identification (surname of first author and year of publication; if more than one paper was present for that author, papers were numbered in brackets following the year of the study), model used, intervention, the number of models used, the survival measurement, the mean biochemical, histological and behavioral measurements and whether the intervention was efficacious for each of these measures was also recorded. Biochemical outcomes included any molecular tests to identify biochemical differences, such as Western blot performed to detect levels of proteins, or markers of autophagy. Histological outcomes included data derived from tissue sections (frozen or formalin fixed, paraffin embedded), for example *in situ* hybridization or immunohistochemistry. Behavioral outcomes included any behavior in animals, e.g., locomotion or maze activity. The timing of the intervention, if stated, was categorized into one of four categories: (i) prior to symptom onset, (ii) at symptom onset, (iii) after symptom onset, or (iv) end-stage of disease. Finally, the quality of each study was categorized based on the number of checklist items scored from a modified version of the CAMARADES quality checklist including: peer review publication; statement of potential conflict of interests; sample size calculation; random allocation to treatment; allocation concealment; blinded assessment of outcome; appropriate control group identified; compliance with animal welfare regulations; statement of temperature control.

### Data Analysis

Survival/mortality data extracted from the included papers were included on a forest plot using the freely available Review Manager (RevMan 5.0) software. Given the variety of model organisms included in the analysis, we expressed effect sizes for the primary outcome data (survival summary data) as odds ratios (OR; Vesterinen et al., 2014). For non-survival outcomes standardized mean difference (SMD) was used. This allowed us to compare the magnitude of the effect size rather than absolute effect sizes, meaning that all data were on the same scale irrespective of the animal model used. The OR/SMD were included on a forest plot using Review Manager 5 (RevMan5.0) software. We calculated summary estimates using a random effects model using the RevMan 5.0 software, weighted by study size, using Hedges g statistic to account for bias from small sample sizes. We reported heterogeneity using *I*^2^ values and used a funnel plot to assess for the presence of publication bias. Predetermined subgroup analyses were performed on compounds tested in three or more separate studies (rapamycin and arimoclomol and lithium were the only three compounds to fit this *a priori* criterion). Other predefined subgroup analyses included an assessment of timing of intervention and quality of studies, where studies were grouped by the (i) timing of the intervention tested (pre-symptom onset, at symptom onset, after symptom onset, end-stage disease) or (ii) quality score and the number of studies showing efficacy were compared between groups using a 2-way ANOVA.

## Results

**Figure 1:**
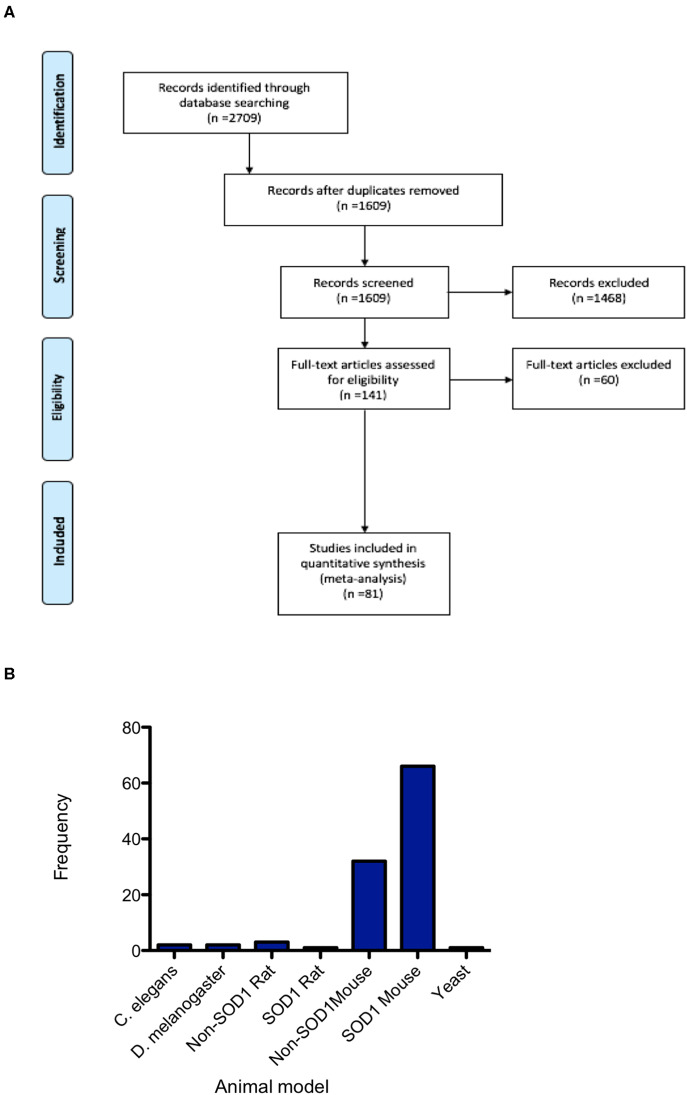
PRISMA flowchart. **(A)** PRISMA Flow Diagram The PRISMA Group (2009) indicating numbers of studies at each stage of the review. **(B)** Frequency distribution summarizing the number of studies conducted in each of the animal models, demonstrating the majority of studies are conducted in mice and predominantly in the SOD1 G93A mouse model.

Database searching identified 2,709 papers meeting the search criteria, which after duplicate removal, resulted in 1,609 articles that underwent screening (Figure 1). Abstracts and titles of articles were screened by three independent screeners using the online SyRF facility resulting in 141 articles that met the predefined inclusion criteria. Following full-text retrieval only 81 of these articles were included in the quantitative analysis (Figure 1A). The majority of animal models investigated were mouse models, with particular emphasis on the *SOD1 G93A* mutant mouse model. Other models used included rat, *Drosophila, C. elegans* and yeast (Figure 1B).

### Targeting Protein Misfolding Confers a Statistically Significant Improvement of Survival

**Figure 2:**
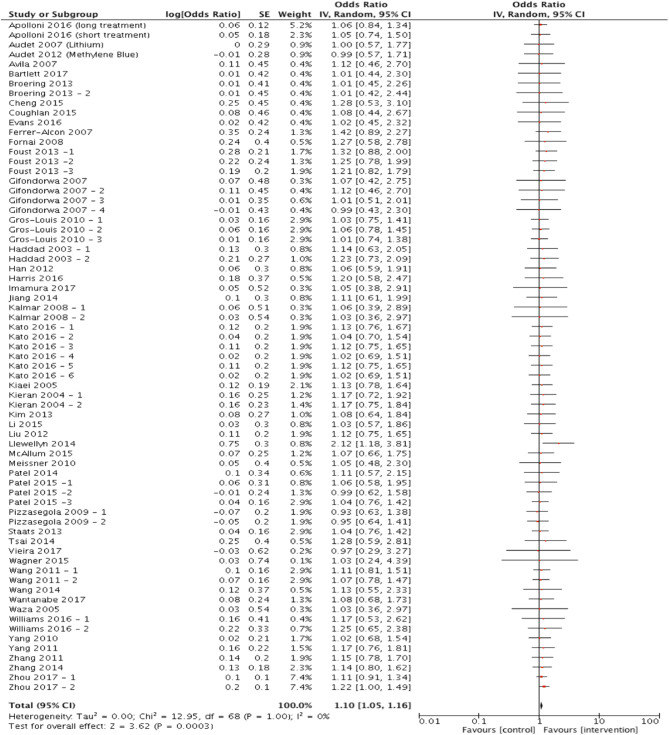
Meta-analysis of preclinical studies shows therapeutic potential for targeting proteostasis in ALS. Forest plot showing the odds ratio and confidence intervals calculated from survival summary data study from each study, weighted by study size. Overall effect estimate is demonstrated (with 95% confidence intervals) as a black diamond at the bottom of the graph. Heterogeneity is displayed as an *I*^2^ value. Results demonstrate an overall statistically significant effect favoring the targeting of proteostasis in ALS.

Out of the 81 articles which met the eligibility criteria, 70 studies assessed survival/mortality as an outcome measure and were included in the meta-analysis to assess the hypothesis that targeting protein misfolding improves survival in ALS/FTD preclinical models. Targeting protein misfolding did significantly extend survival compared to untreated controls (*p* < 0.001, degrees of freedom (df) = 68, α = 0.05 and 95% confidence intervals (CI) 1.05-1.16; Figure 2). The heterogeneity of these included studies was estimated by calculating an *I*^2^ value. The *I*^2^ value in this analysis was 0%, indicating a high level of agreement between studies favoring treatment over control (Figure 2).

### Timing of Intervention Favors Early Treatment

**Figure 3:**
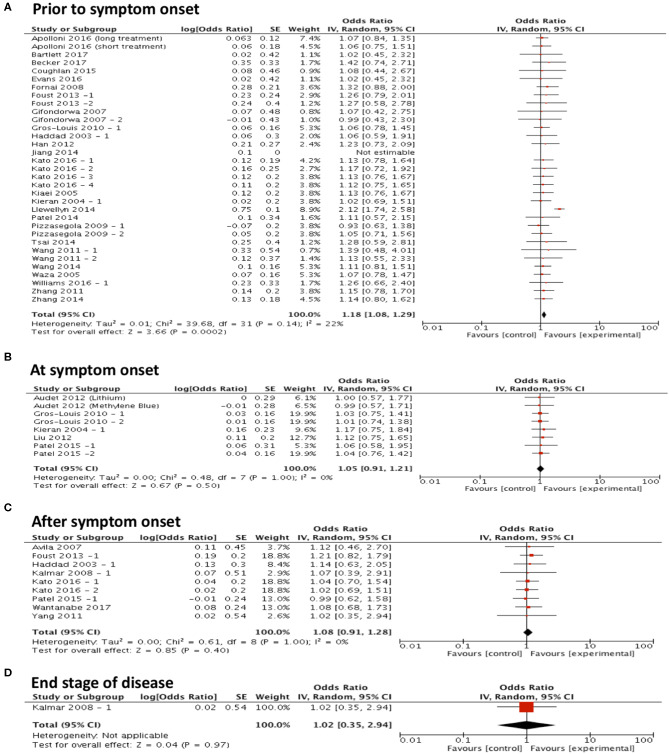
Majority of studies are conducted at early time points in ALS research and show that with early interventions there is a statistically significant improvement in survival. Forest plots showing the odds ratio and confidence intervals calculated from survival summary data study from each study, weighted by study size. Overall effect estimate is demonstrated (with 95% confidence intervals) as a black diamond at the bottom of the graph. Heterogeneity is displayed as an *I*^2^ value. **(A)** Intervention delivered pre-symptom onset; **(B)** at symptom onset and **(C)** after symptom onset. **(D)** At end stage of disease. Results demonstrate an overall statistically significant effect favoring the targeting of proteostasis early in ALS **(A)**.

To assess the hypothesis that targeting protein misfolding at different stages of the disease process will affect the efficacy of treatments on survival, studies were included in a forest plot grouped by timing of intervention and repeat sub-group meta-analyses were performed (Figures 3A–D). These analyses found that only treating prior to symptom onset (*n* = 33) significantly improved survival (*p* < 0.001, df = 31, α = 0.05 and CI = 1.08–1.29; Figure 3A). No significant improvement in survival was found when treating at symptom onset (*p* = 0.50, α = 0.05, df = 7, *n* = 8, and CI = 0.91–1.21; Figure 3B), after symptom onset (*p* = 0.40, α = 0.05, df = 8, *n* = 9, and CI = 0.91–1.28; Figure 3C) or at the end stage of disease (*p* = 0.97, α = 0.05, df = N/A, *n* = 1, and CI = 0.35–2.94; Figure 3D). Although it should be noted that there are considerably fewer studies conducted at these later time points.

### Arimoclomol and Lithium Demonstrate Therapeutic Potential in Preclinical Studies

**Figure 4:**
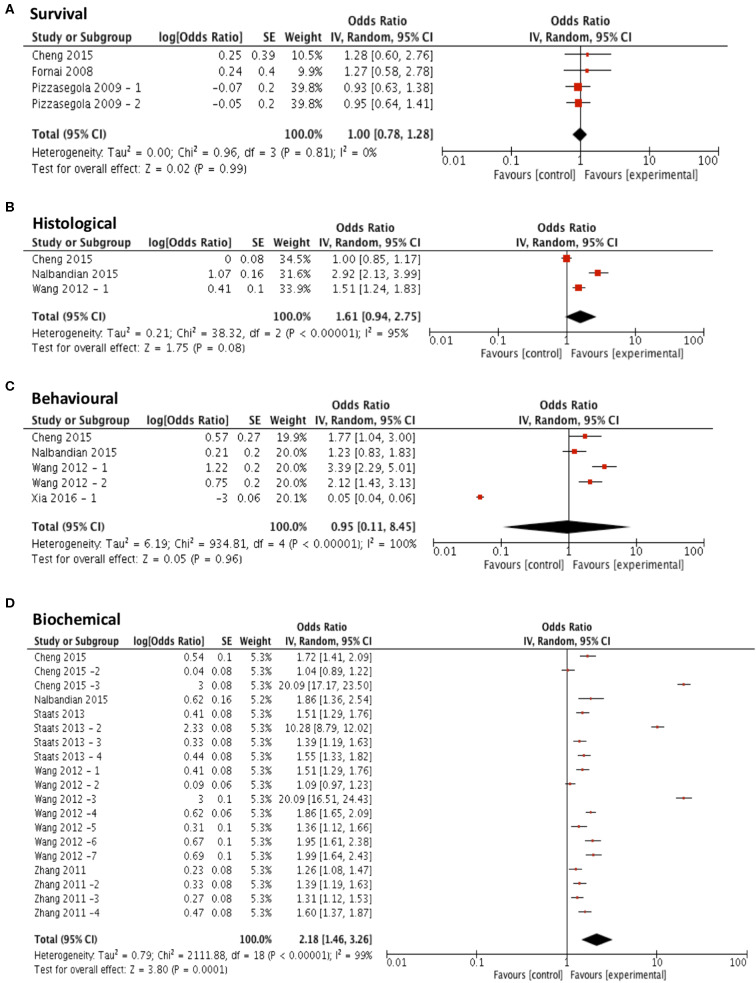
Rapamycin does not demonstrate efficacy in targeting survival, behavioral or histological outcomes. Forest plots showing the odds ratio (for survival) and SMD (for histological, behavioral and biochemical outcomes) and confidence intervals calculated from each study, weighted by study size. Overall effect estimate is demonstrated (with 95% confidence intervals) as a black diamond at the bottom of the graph. Heterogeneity is displayed as an *I*^2^ value. **(A)** Studies assessing the effect of rapamycin on survival (*n* = 4). **(B)** Studies assessing the effect of rapamycin on histological outcomes (*n* = 3). **(C)** Studies assessing the effect of rapamycin on behavioral outcomes (*n* = 5). **(D)** Studies assessing the effect of rapamycin on biochemical outcomes (*n* = 19). Results demonstrate an overall statistically significant effect only on biochemical outcome measures when treating with rapamycin **(D)**.

Three compounds met our predefined subgroup analysis criteria having been investigated 3 or more times in independent studies; rapamycin, arimoclomol, and lithium. Studies investigating these compounds were evaluated by meta-analysis against both primary and secondary outcome measures. Subgroup analyses revealed that rapamycin treatment (*n* = 4) did not significantly improve survival compared to control (*p* = 0.99, df = 3, α = 0.05, and CI = 0.78–1.28; Figure 4A). Furthermore, meta-analyses assessing the efficacy of rapamycin treatment on secondary outcome measures revealed that it did not significantly reduce histological outcomes (*p* = 0.08, df = 2, α = 0.05, and CI = 0.94–2.75; Figure 4B) or behavioral outcomes (*p* = 0.96, df = 4, α = 0.05, and CI = 0.11–8.45; Figure 4C). However, rapamycin treatment did significantly affect biochemical outcomes (*p* < 0.001, df = 18, α = 0.05, and CI = 1.46-3.26; Figure 4D). The heterogeneity of the studies investigating survival was 0%.

**Figure 5:**
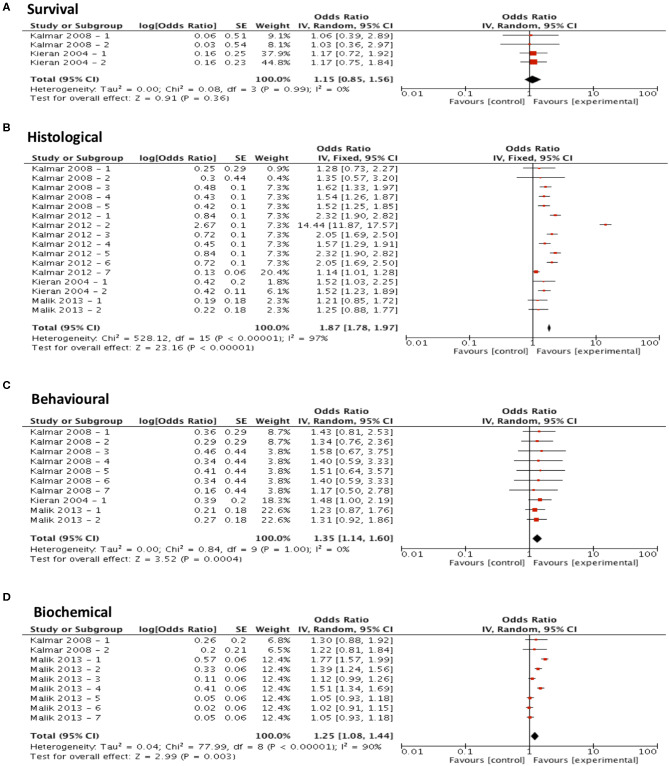
Arimoclomol demonstrates potential in modifying all secondary outcome measures tested. Forest plots showing the odds ratio (for survival) and SMD (for histological, behavioral and biochemical outcomes) and confidence intervals calculated from each study, weighted by study size. Overall effect estimate is demonstrated (with 95% confidence intervals) as a black diamond at the bottom of the graph. Heterogeneity is displayed as an *I*^2^ value. **(A)** Studies assessing the effect of arimoclomol on survival (*n* = 4). **(B)** Studies assessing the effect of arimoclomol on histological outcomes (*n* = 16). **(C)** Studies assessing the effect of arimoclomol on behavioral outcomes (*n* = 10). **(D)** Studies assessing the effect of arimoclomol on biochemical outcomes (*n* = 9). Results demonstrate an overall statistically significant effect only on all secondary outcome measures when treating with arimoclomol **(B–D)**.

Meta-analysis assessing the efficacy of arimoclomol treatment on survival (*n* = 4) revealed no significant improvement in survival (*p* = 0.36, df = 3, α = 0.05, and CI = 0.85-1.56; Figure 5A) but did show significant improvements in all secondary outcome measures including histological outcomes (*p* < 0.001, df = 15, α = 0.05, and CI = 1.78–1.97; Figure 5B), behavioral outcomes (*p* < 0.001, df = 9, α = 0.05, and CI = 1.14-1.60; Figure 5C) and biochemical outcomes (*p* = 0.003, df = 8, α = 0.05, and CI = 1.08–1.44; Figure 5D). The heterogeneity of the studies investigating survival and behavioral measures was 0% suggesting a high level of agreement between studies. However, evaluation of heterogeneity when assessing histological and biochemical measurements revealed high levels of heterogeneity (*I*^2^ > 50).

**Figure 6:**
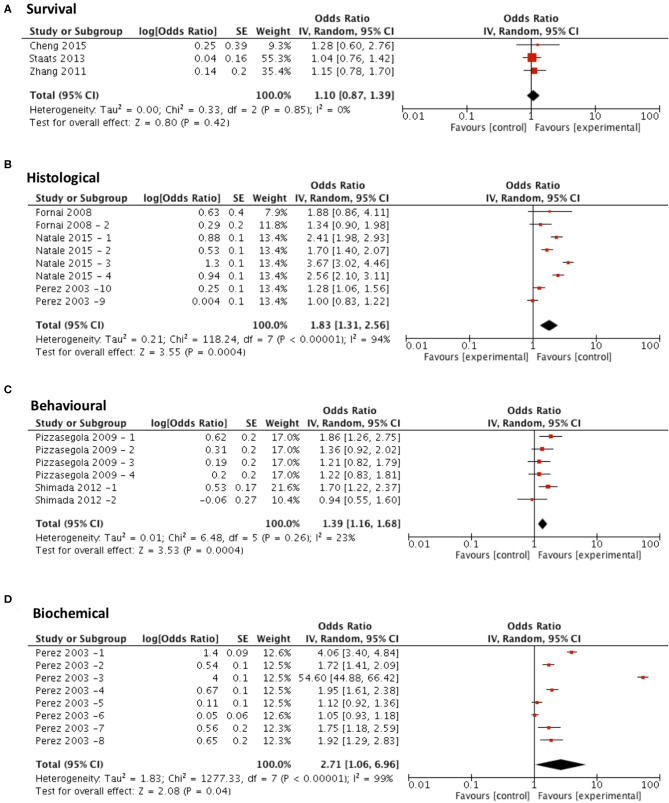
Lithium demonstrates potential in modifying all secondary outcome measures tested. Forest plots showing the odds ratio (for survival) and SMD (for histological, behavioral and biochemical outcomes) and confidence intervals calculated from each study, weighted by study size. Overall effect estimate is demonstrated (with 95% confidence intervals) as a black diamond at the bottom of the graph. Heterogeneity is displayed as an *I*^2^ value. **(A)** Studies assessing the effect of lithium on survival (*n* = 3). **(B)** Studies assessing the effect of lithium on histological outcomes (*n* = 8). **(C)** Studies assessing the effect of lithium on behavioral outcomes (*n* = 6). **(D)** Studies assessing the effect of lithium on biochemical outcomes (*n* = 8). Results demonstrate an overall statistically significant effect only on all secondary outcome measures when treating with lithium **(B–D)**.

Meta-analysis assessing the efficacy of lithium treatment on survival (*n* = 3) revealed no significant improvement in survival (*p* = 0.42, df = 2, α = 0.05, and CI = 0.87–1.39; Figure 6A) but did show significant improvements in all secondary outcome measures including histological outcomes (*p* = 0.0004, df = 7, α = 0.05, and CI = 1.31–2.56; Figure 6B), behavioral outcomes (*p* = 0.0004, df = 5, α = 0.05, and CI = 1.16–1.68; Figure 6C) and biochemical outcomes (*p* = 0.04, df = 7, α = 0.05, and CI = 1.06–6.96; Figure 6D). The heterogeneity of the studies investigating survival and behavioral measures was 0 and 23% respectively, suggesting a high level of agreement between studies. However, evaluation of heterogeneity when assessing histological and biochemical measurements revealed high levels of heterogeneity (*I*^2^ > 50).

### Overall Quality of Studies Did Not Affect Likelihood of Efficacy

**Figure 7:**
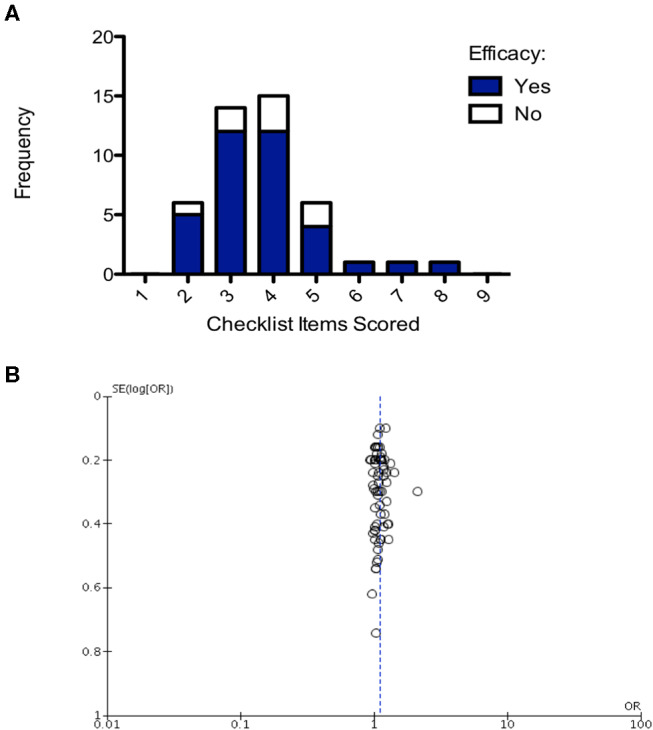
Structured quality assessment shows no demonstrable effect on study efficacy and no evidence of publication bias. **(A)** Frequency distribution demonstrating that the structured quality score is not significant in determining intervention efficacy (2-way ANOVA *p* > 0.05). **(B)** Funnel plot, each point is a study, plotted against the effect size of that study (x-axis) and precision of that study (SE(log[OR]); y-axis). Publication bias has not been detected in this analysis.

Number of checklist items scored was evaluated for all studies and then plotted on a frequency distribution to evaluate the likelihood of those studies demonstrating efficacy. The hypothesis was that poorer quality studies would score fewer checklist items and result in inappropriate demonstrations of efficacy (i.e., false positive/false negative). The two-way ANOVA comparing frequency of efficacy in the studies of different quality and the *post-hoc* Bonferroni test accounting for multiple testing found there was no significant difference in the frequency of efficacy across differences in study quality (*p* = 0.118; Figure 7A). Finally, to assess whether there was publication bias in studies included in this review a funnel plot was created showing one outlier study, but no evidence of publication bias (Figure 7B).

## Discussion

### Targeting Protein Misfolding Confers Significant Survival Improvement

The results of this study support the hypothesis that protein misfolding plays a major role in the pathogenesis of ALS and FTD. Meta-analysis of 70 studies (assessing survival outcomes) identified that targeting protein misfolding significantly extended survival compared to untreated controls with minimal heterogeneity detected between studies (*I*^2^ = 0). A list of the interventions that were tested in these studies, including the putative mechanism of action of those interventions has been provided in Table 1. Further analysis of interventions investigated in at least three independent studies identified that rapamycin did not confer an improvement in either survival or secondary outcome measures (histological or behavioral). The mammalian target of rapamycin (mTOR) is a ubiquitously expressed kinase with a key role in mediation of major processes such as; metabolism, immunoregulation and the inhibition of autophagy. Rapamycin belongs to a class of drugs which inhibit mTOR activity, the pro-autophagic effect of these drugs has been explored in animal models of degenerative proteinopathic disease such as Alzheimer's disease (Dolcetta and Dominici, 2019). The neuroprotective role of mTOR has also been identified in SOD1 mouse models of ALS as a key mechanism to prevent motor neuron degeneration and disease progression (Saxena et al., 2013). One possible explanation for our study findings may be the development of tolerance which has been identified previously after continuous administration of an mTOR inhibitor resulted in pathway hyper-stimulation and an initial short-term stimulation of autophagy preceded by a longer-term decrease in autophagy markers (Kurdi et al., 2016). Another potential mechanism to explain the broader inconsistency in mTOR inhibition efficacy in ALS models (Zhang et al., 2011) is the observation in AD models of impaired autophagosome clearance, as the pro-autophagic effects would be counteracted by the accumulation of these autophagosomes (Bove et al., 2011). The small sample size included in the secondary meta-analysis of rapamycin may also limit the ability to reach definitive conclusions.

**Table 1:** List of interventions tested and putative pathways implicated.

**Intervention tested**	**Putative pathway**
Clemastine	Protein clearance (autophagy)
Trichostatin A	Protein clearance (autophagy)
Methylene Blue	Chaperone activity
Lithium	Protein clearance (autophagy)
Dimebon	Chaperone activity
Brilliant blue G	Reduction in neuroinflammation (inhibits P2RY receptor) therefore reduction in ER stress
Rapamycin	Protein clearance (autophagy)
Latrepirdine	Physical modulation of misfolded proteins and blockade of calcium channels reducing excitotoxicity and subsequent ER stress
Recombinant Hsp70	Chaperone activity
Riluzole	Chaperone activity (modulates HSF1 expression)
Resveratol	ER stress and autophagy
Follistatin	Protein clearance (autophagy)
Bosutinib	Protein clearance (autophagy)
Guanabenz	ER stress
Arimoclomol	Chaperone activity
Celastrol	Chaperone activity
Progesterone	Chaperone activity
Allopurinol	Autophagy and ER stress
Trehalose	Autophagy and ER stress
Zinc	Chaperone activity
Withaferin A	Ubiquitin proteasome system
17-AAG (geldanamycin)	Autophagy and Chaperone activity
Cystatin C	Physical modulation of and binding to misfolded proteins and autophagy
Copper	Chaperone activity
Melittin (bee venom)	Autophagy and ER stress

*A table listing the therapeutic interventions analyzed as part of this systematic review including the putative pathway implicated in the mechanism of action. It is important to note however that drugs can modulate multiple potential targets, and that the dogma of ‘single drug, single target' is overly simplistic. This table is provided to give an overview of the interventions tested with information of a putative target with respect to its role in proteostasis, noting that many of these interventions will have other target effects not listed here*.

Arimoclomol, a hydroxylamine derivative is a heat shock protein (HSP) co-inducer and promotor of native protein folding, the finding that arimoclomol treatment demonstrated an improvement in behavioral outcomes may indicate that it could be implemented as a promising therapeutic aimed at alleviating symptoms and improving quality of life in ALS (Dairin et al., 2004). However, it is worth noting that arimoclomol had no effect on survival outcomes. Importantly, arimoclomol, as a co-inducer rather than an activator can stimulate HSP network upregulation within cells that are already stressed. This selectivity is important mechanistically to prevent imbalances caused by single HSP upregulation (for example selective Hsp90 or Hsp70 induction) and also to target the HSPs only where required (Kalmar et al., 2008). Indeed the potential therapeutic benefit of arimoclomol has also been suggested by a recent phase II clinical trial including a cohort of patients with rapidly progressive SOD1 ALS (Benatar et al., 2018).

The diverse neuro-protective effects of lithium have been explored in preclinical *in vivo* models of dementia and are attributed to a range of mechanisms including oxidative stress, neuro-inflammation, cell survival, neurogenesis, synaptic plasticity and proteostasis. The main regulatory pathways are via inhibition of inositol monophosphatase (IMPA) and glycogen synthase kinase-3 (GSK-3), however multiple direct interactions with downstream key mediators have also been identified (Kerr et al., 2017). While inhibition of IMPA results in the mTOR pathway-independent induction of autophagy, the inhibition of GSK-3 results in modulation of multiple neuroprotective mediators including Nrf2 and STAT3 (Kerr et al., 2018). GSK-3β inhibition also has the therapeutically undesirable effect of activating the mTOR pathway and attenuating autophagy (Sarkar et al., 2008). The resultant opposing mTOR independent/dependent effects on autophagy may reflect, in part, why attempts to assess efficacy in *SOD1G93A* mice treated with lithium mono-therapy have produced conflicting results (Fornai et al., 2008; Gill et al., 2009; Pizzasegola et al., 2009). To address the therapeutic limitation of lithium mediated GSK-3β inhibition and resultant m-TOR activation, a targeted strategy using combination therapy with lithium and rapamycin has been assessed in a Huntington's disease *in vivo* model demonstrating an additive neuroprotective effect (Sarkar et al., 2008). The combined approach of selectively manipulating separate pathways simultaneously has been extended to the use of lithium coupled with valproate and also a novel antioxidant, in both studies concurrent administration resulted in improved motor function and survival in *SOD1G93A* mice (Shin et al., 2007; Feng et al., 2008). Furthermore, daily lithium treatment co-administered with riluzole has been identified to reduce disease progression in a study of ALS patients followed up over 15 months (Fornai et al., 2008).

### Timing of Intervention

No significant improvement in survival was found when treating at or after symptom onset, further analysis identified that only treatment with therapies targeting protein misfolding prior to symptom onset significantly improved survival. While this is in keeping with the neurodegenerative model; to treat before the accumulation of higher stability neurotoxic protein aggregates (Glickman and Ciechanover, 2002), the practical application of this to rapidly progressive diseases such as ALS presents a major challenge. This raises the issue of genetic screening within a genetically heterogeneous, variably penetrant disease. Interestingly, the delivery of arimoclomol after symptom onset and even at late-symptomatic disease stage has been found to improve muscle function in a SOD1 mouse model, it has been suggested that this reflects the stress-dependent mechanism of action of arimoclomol. In this study, an increase in lifespan was however only observed in treated mice from symptom onset (Kalmar et al., 2008). Therapies designed to target different proteostatic subsystems are likely to have differing windows of optimal efficacy, for this reason the timing of therapy initiation is a critical factor for further therapeutic research. Furthermore, the small sample size of studies conducted at later time points is a major limitation of the field of neurodegenerative diseases and is likely impacting upon our ability to assess these time points with confidence in our study. Future work should aim to address this in the field with studies conducted at later time points (especially after symptom onset) being more relevant to our ability to effectively translate these interventions to the clinic.

### Limitations

A key limitation of studies of this nature is that study identification is based on a statement by the authors that the intervention was tested on protein misfolding pathways. We specifically employed this method to minimize bias and to identify a comprehensive dataset, aware that drugs rarely have a single mode of action, to enable us to study drugs where their commonality is an effect on proteostasis, rather than their sole effect. We note that no approach to identify such interventions is perfect and if a study did not mention an action on proteostasis, we were not able to include it in our analysis.

Furthermore, the majority of papers identified in this study are carried out in the mutant *SOD1* rodent model. Whilst this is a well-established animal model used widely in the field, it does not necessarily model the most common pathology seen in ALS patients, which is the misfolding and mislocalisation of TDP-43. Whilst many pathological consequences of proteostasis may be shared between these misfolded proteins, wider use of other animal models assessing the effects of modulating proteostasis on other misfolded proteins is clearly warranted.

As anticipated, the heterogeneity of the dichotomous primary outcome measure, survival, was consistently low. In contrast, biochemical and histological outcomes (continuous rather than dichotomous outcome measures) demonstrate varying levels of heterogeneity, ranging from 0–100% across the three therapies assessed in our subgroup analyses. This finding of higher levels of heterogeneity with continuous outcomes measures is consistent with the literature and for this reason it has been suggested that different standards should be applied to the interpretation of *I*^2^ for each (Alba et al., 2016). Where large variability in effect estimates across continuous measures is observed, the consistency in the direction of the effect is also a relevant factor to consider.

We did not detect any publication bias in the studies included in this review. Publication bias can result in effect size estimates being skewed to find a greater effect than is true due to positive results being favored for publishing compared to negative results. Indeed, the Amyotrophic Lateral Sclerosis Therapy Development Institute found that on average, studies report significantly greater effects of therapeutics on extending survival than is true when compared to rigorous testing in controlled laboratory conditions with conscious efforts to reduce the risk of unconscious bias. This is thought to have contributed, in part, to disappointing translation when these therapeutics have been trialed in humans. These studies, taken together with our data indicate that we should exercise caution in interpreting preclinical studies and be aware that they could reflect an overestimate of the true effect size. This highlights the importance of quality assessments and risk of bias scoring done as a part of systematic reviews such as ours. However, measures of quality, like the CAMARADES checklist, do not necessarily reflect the importance of each quality control measure. For example, lack of experimental blinding may affect the efficacy and/or reproducibility of results more than peer review. This means that even papers that have scored the highest number of checklist items in our analysis are not achieving all of the necessary quality control steps to improve reliability of results. The limited number of high quality studies may also explain why results are often overstated in the field because if fundamental quality control is not implemented results can be unreliable and this is likely to contribute to poor clinical translation of potential therapeutics.

### Implications and Recommendations for Future Research

Protein misfolding, as a pathological entity, links many diverse neurodegenerative diseases which have previously been considered to be distinct clinical entities and may even contribute to normal aging. The risk of pathological protein misfolding can be increased by environmental stressors and disease-causing mutations and the innate proteostatic mechanisms to counter this are known to be compromised by aging (Kampinga and Bergink, 2016; Kalmar and Greensmith, 2017). Therefore, it is plausible that targeting of this common pathophysiology, irrespective of the underlying genetic basis of the condition, may be therapeutically beneficial for many other currently untreatable neurodegenerative diseases.

## Conclusions

This is the first study to formally assess the therapeutic potential of targeting protein misfolding in ALS and FTD and to perform a structured quality of assessment of the ALS and FTD preclinical literature. Our data support the hypothesis that protein misfolding contributes to pathology in ALS/FTD. We show that targeting protein misfolding in ALS/FTD significantly improves survival however, treatments only conclusively demonstrate efficacy when administered prior to symptom onset. This limits the clinical potential of these therapies targeting protein misfolding, as genetic screening and pre-treatment of patients has many ethical implications. Notwithstanding the potential risk of bias owing to overestimation of effect sizes and risk of bias between studies, we identified promising therapeutics, such as arimoclomol and lithium, which demonstrated a statistically significant improvement in histological, behavioral and biochemical outcome measures. Further investigations should be aimed at identifying specific therapeutics that target protein misfolding and improve symptoms or survival, so they can be developed for clinical trials to treat ALS/FTD and future studies should employ multiple therapeutic intervention timepoints to improve clinical translation in the future.

## Data Availability Statement

All data have been made available within the publication material.

## Author Contributions

OB, FW and JG contributed to manuscript screening and data extraction. All authors contributed to conceptualisation and experimental design, data interpretation, and manuscript authorship.

## Conflict of Interest

The authors declare that the research was conducted in the absence of any commercial or financial relationships that could be construed as a potential conflict of interest.
